# Supplementary material for: Estimating the time-varying effective reproduction number via Cycle Threshold-based Transformer
Source: PLoS Comput Biol. 2024 Dec 23;20(12):e1012694. doi: 10.1371/journal.pcbi.1012694 (PMC11706484; doi:10.1371/journal.pcbi.1012694)
Supplement: S1 Supplementary Methods — 1. Parameters of the Agent-based SEIR Transmission Model and Ct Value Model. 2. Calculation of Rt based on Micro-transmission Chains. 3. Ct Variables in the Synthetic Datasets. 4. Hyper-parameters of Deep Learning Methods. 5. Confidence Intervals of Rt Estimation. 6. Further Exploration of Detection Rate on Model Performance. 7. Rate of Masked Patches for Self-supervised Learning. 8. Analysis of the Patching Layer. (ZIP) [file pcbi.1012694.s001.zip › S1_Supplementary Methods.pdf]

Estimating the time-varying effective reproduction number via Cycle Threshold-based Transformer

Xin-Yu Zhang[1,2], Lan-Lan Yu[1,2], Wei-Yi Wang[1,2], Gui-Quan Sun[3,4*], Jian-Cheng Lv[1,2], Tao Zhou[5], Quan-Hui Liu[1,2*],

1 College of Computer Science, Sichuan University, Chengdu, China
2 Engineering Research Center of Machine Learning and Industry Intelligence, Ministry of Education, Sichuan University, Chengdu, China
3 Department of Mathematics, North University of China, Taiyuan, China
4 Complex Systems Research Center, Shanxi University, Taiyuan, China
5 Big Data Research Center, University of Electronic Science and Technology of China, Chengdu, China

* Corresponding authors: gquansun@126.com (G.-Q. S.); quanhuiliu@scu.edu.cn (Q.-H. L.)

Supplementary Methods

1 Parameters of the Agent-based SEIR Transmission Model and Ct Value Model 2

2 Calculation of R_t based on Micro-transmission Chains 3

3 Ct Variables in the Synthetic Datasets 5

4 Hyperparameters of Deep Learning Methods 6

5 Confidence Intervals of R_t Estimation 7

6 Further Exploration of Detection Rate on Performance 8

7 Rate of Masked Patches for Self-supervised Learning 9

8 Analysis of the Patching Layer 10

1 Parameters of the Agent-based SEIR Transmission Model and Ct Value Model

In the SEIR transmission model [1], before initiating the simulation of transmission, we establish a total of N_{total} individuals, among which N_{seed} individuals are set to an initial state of I. When susceptible individuals come into contact with their infected neighbors, they have a probability β of being exposed $(S \rightarrow E)$, which is calculated as $\beta = R_0 \times \frac{\gamma}{\langle k \rangle}$. Following a period known as the latent period ε, exposed individuals begin to show symptoms and become infectious $(E \rightarrow I)$. They then experience an infected period ω and eventually recover $(I \rightarrow R)$.

In the Ct value model [2], the trajectory of Ct values for each infected individual since the infection can be expressed as the Eq. (S1), where $Ct(a)$ denotes the Ct value after a days of infection. These trajectories of Ct values are shown in S1 Fig. By keeping the infection time for each infected individual in the micro-chain of transmission and this Ct value model, we can produce the Ct values of all infected individuals at each time step during the period of outbreak. All the parameters about the SEIR transmission model and Ct value model are listed in S1 Table.

$$Ct(a) = \begin{cases} C_{zero}, a \leq t_{eclipse} \\ C_{zero} + \frac{t_{peak}}{C_{peak} - C_{zero}}(a - t_{eclipse}), t_{eclipse} < a \leq t_{eclipse} + t_{peak} \\ C_{peak} + \frac{t_{end}}{C_{zero} - C_{peak}}(a - t_{eclipse} - t_{peak}), t_{eclipse} + t_{peak} < a \leq t_{eclipse} + t_{peak} + t_{end} \end{cases} \quad \text{(S1)}$$

S1 Fig. The trajectories of Ct values. The gray lines represent the trajectories of Ct values for 50 randomly selected infected individuals, while the black line represents the trajectory of one infected individual.

S1 Table. Parameters of the Agent-based SEIR Transmission Model and Ct Value Model.

2 Calculation of R_t based on Micro-transmission Chains

We keep all micro-transmission chains when modeling the epidemic spreading using the algorithm described in S1 Algorithm. Before simulating the epidemic spreading, we initialize the incubation period ε, infectious period ω of each individual by randomly sampling from the Poisson distribution. All individuals are in susceptible state, with N_{seed} individuals are set to an initial state of I and added to the list $\mathcal{Q}^I_{old}$. Numerical calculations are then performed at each time step t during the simulation. In specific, if the list $\mathcal{Q}^I_{old}$ and $\mathcal{Q}^E_{old}$ are not empty, we traverse the individuals in $\mathcal{Q}^I_{old}$. If an infection occurs on a neighboring individual, add the newly infected neighboring individual to $\mathcal{Q}^E_{new}$. Once the transmission from each individual in $\mathcal{Q}^I_{old}$ is completed at time step t, we update the status of all individuals as follows: We first traverse $\mathcal{Q}^E_{old}$. If an individual remains in exposed state, we add it to $\mathcal{Q}^E_{new}$. If the state changes to infectious state, we move it to $\mathcal{Q}^I_{new}$. We finally swap $\mathcal{Q}^E_{new}$ with $\mathcal{Q}^E_{old}$. Then we traverse the $\mathcal{Q}^I_{old}$, if an individual remains in infectious state, we add it to $\mathcal{Q}^I_{new}$. We finally Swap $\mathcal{Q}^I_{new}$ with $\mathcal{Q}^I_{old}$ and advance the simulation to the next time step $t+1$.

Throughout this process, we record the number of secondary infections $\Omega_{(v)}$ for each v and the nodes Φ_t infected at time t to construct the micro-transmission chains. We calculate R_t directly according to the definition of the time-varying effective reproduction number R_t [3].

S1 Algorithm The agent-based SEIR transmission process and the calculation of time series of R_t

Input:

N_{total}: the number of nodes;
$\mathcal{N}_v$: nodes in contact with node v;
N_{seed}: initial number of infected seeds; $N_{seed} \leftarrow 1$
$\mathcal{Q}^E_{old}(\mathcal{Q}^E_{new})$: the list to record the old (new) exposed nodes; $\mathcal{Q}^E_{old} \leftarrow \varnothing$; $\mathcal{Q}^E_{new} \leftarrow \varnothing$
$\mathcal{Q}^I_{old}(\mathcal{Q}^I_{new})$: the list to record the old (new) infectious nodes; $\mathcal{Q}^I_{old} \leftarrow \varnothing$; $\mathcal{Q}^I_{new} \leftarrow \varnothing$
$\Omega_{(v)}$: record the number of secondary infections caused by node v; $\Omega_{(v)} \leftarrow 0$
Φ_t: the list to record the nodes infected at time t; $\Phi_t \leftarrow \varnothing$
$t \leftarrow 1$
for each $v \in \mathcal{V}$ **do**
  Sample incubation period ε from the incubation period distribution for node v
  Sample infectious period ω from the infectious period distribution for node v
end for
Randomly add N_{seed} seed nodes to $\mathcal{Q}^I_{old}$ and Φ_t
while $\mathcal{Q}^I_{old} \neq \varnothing$ and $\mathcal{Q}^E_{old} \neq \varnothing$ **do**
  $t \leftarrow t + 1$
  for each v in $\mathcal{Q}^I_{old}$ **do**
    for each u in $\mathcal{N}_v$ **do**
      if infection occurs **then**
        Add u to $\mathcal{Q}^E_{new}$ and Φ_t; $\Omega_{(v)} \leftarrow \Omega_{(v)} + 1$
      end if
    end for
  end for
  for each v in $\mathcal{Q}^E_{old}$ **do**
    if v enters infectious period **then**
      Add v to $\mathcal{Q}^I_{new}$
    else
      Add v to $\mathcal{Q}^E_{new}$
    end if
  end for
  Swap $\mathcal{Q}^E_{old}$ and $\mathcal{Q}^E_{new}$; $\mathcal{Q}^E_{new} \leftarrow \varnothing$
  for each v in $\mathcal{Q}^I_{old}$ **do**
    if v does not enter recover period **then**
      Add v to $\mathcal{Q}^I_{new}$
    end if
  end for
  Swap $\mathcal{Q}^I_{old}$ and $\mathcal{Q}^I_{new}$; $\mathcal{Q}^I_{new} \leftarrow \varnothing$
end while
$L \leftarrow t$
for $t = 1$ to L **do**
  $A_t \leftarrow 0$
  for each v in Φ_t **do**
    $A_t \leftarrow A_t + \Omega_{(v)}$
  end for
  $R_t \leftarrow A_t / |\Phi_t|$
end for

Output:

Time series of R_t of length L, $[R_1, R_2, ..., R_L]$

3 Ct Variables in the Synthetic Datasets

We explore the impact of inputs with different Ct variables in the synthetic datasets on the Ct-Transformer performance, including the average, skewness and distribution of Ct values. The average ($\bar{x_t}$) and skewness (b_t) of Ct values at time step t are respectively calculated as:

$$\bar{x_t} = \frac{1}{n_t}\sum_{i=1}^{n_t} y_{t,i}, \tag{S2}$$

$$b_t = \frac{\frac{1}{n_t}\sum_{i=1}^{n_t}(y_{t,i} - \bar{x_t})^3}{[\frac{1}{n_t}\sum_{i=1}^{n_t}(y_{t,i} - \bar{x_t})^2]^{\frac{3}{2}}} \tag{S3}$$

where $y_{t,i}$ represents the $i-$th $(i = 1, 2, ..., n_t)$ of the total n_t Ct values that are sampled at time step t.

To deal with the continuous Ct values, which range from 16 to 40, we define the set $D = \{D_1, D_2, D_3, ..., D_d\}$ for the categories used to calculate the probability distribution. The distribution of Ct values is formatted as $[P(Ct_t)_{D_1}, P(Ct_t)_{D_2}, ..., P(Ct_t)_{D_d}]$. Therein, $P(Ct_t)_{D_i}$ represents the proportion of infected individuals with Ct values falling in the D_i interval at time step t. The possible scenarios for the number of intervals d and the corresponding set D are listed in S2 Table:

S2 Table. Intervals d and the set D in the distribution of Ct values.

The results of the supervised Ct-Transformer using different categories for the distributions of Ct values are summarized in S3 Table. We compare the performance on each category for the distributions of Ct values and define $D = \{[16, 22], (22, 28], (28, 34], (34, 40]\}$ for the main result.

S3 Table. The sensitivity results of intervals d on ER and SF datasets. The **Average** means the average of simulations with $R_0 \in \{1.2, 1.8, 2.2, 2.8, 3.4\}$ in the testing set. For each R_0 and the **Average**, the best results are in **bold** and the runners-up are presented as underlined.

Further, the results of the supervised Ct-Transformer using different combinations of Ct variables are shown in S4 Table. The combination of the distribution and average of Ct values as the input has the best result for the Ct-Transformer. We use the probability distribution of sampled infected individuals with different Ct values and the average of Ct values as the final input for the Ct-Transformer. The former represents the shape of the distribution of Ct values, while the latter can be proxied as the viral load level of the infected population.

S4 Table. The sensitivity results of Ct variables (Var) on ER and SF datasets. The **Average** means the average of the simulations with $R_0 \in \{1.2, 1.8, 2.2, 2.8, 3.4\}$ in the testing set. For each R_0 and the **Average**, the best results are in **bold** and the runners-up are presented as underlined.

4 Hyperparameters of Deep Learning Methods

We apply a grid search approach to ascertain the optimal hyperparameters for other deep learning methods. The specific tuning spaces of the hyperparameters for all alternative deep learning methods are listed in S5 Table

S5 Table. Hyperparameters, tuning spaces, and the best hyperparameter settings for deep learning methods (Ct-Transformer, TFT, Transformer, and MLP) on the ER dataset and SF dataset.

5 Confidence Intervals of R_t Estimation

We train the Ct-Transformer with quantile loss [4] as defined in the main body. The quantile loss can provide confidence intervals using multiple quantile points. The 95% confidence intervals of R_t estimation calculated using the quantile points $Q = \{0.025, 0.5, 0.975\}$ are shown in S2 Fig. We find that the 95% confidence intervals given by the Ct-Transformer can accurately capture the maximum and minimum scales of the temporal evolution of R_t. The 95% confidence intervals are crucial as they provide governments with the necessary information to be fully prepared for an epidemic.

S2 Fig. The 95% confidence intervals of R_t estimation. The pink line and shaded area respectively represent the average of R_t and the 95% confidence intervals.

6 Further Exploration of Detection Rate on Performance

The full comparisons between the Ct-Transformer and EpiEstim on the SF dataset under different detection scenarios are shown in S6 Table. The supervised Ct-Transformer consistently outperforms the EpiEstim across all detection scenarios, which is consistent with the results on the ER dataset in the main body.

S6 Table. Results of the supervised Ct-Transformer and the EpiEstim method on the SF dataset under different detection scenarios. We show the average results of simulations with $R_0 \in \{1.2, 2.2, 3.4\}$ in the testing set. For each detection scenario, the better one is presented as in **bold**.

In stochastic simulations with R_0=2.2 on both ER and SF networks, the increased percentage in MAE compared to the Full Detection scenarios is shown in S3 Fig and demonstrates minimal for the Ct-Transformer. These results on both ER and SF datasets demonstrate the proposed Ct-Transformer is robust to the time-varying detection rate and limited detection resources.

S3 Fig. The increased percentage in MAE relative to the Full Detection scenario in stochastic simulations on both ER and SF networks with R_0=2.2. The greyish-blue and yellow bars respectively represent the MAE loss of the Ct-Transformer and EpiEstim in different detection scenarios. The numbers displayed on the bars indicate the increased percentage in MAE relative to the Full Detection scenario.

7 Rate of Masked Patches for Self-supervised Learning

We explore the optimal rate of masked patches for self-supervised learning of the Ct-Transformer. In specific, we conduct training process of self-supervised learning on the ER dataset by masking different rates of patches (10%, 20%, 30%, and 40%). These pre-trained models are fine-tuned with the End2End strategy on the SF dataset. The results of different rates of masked patches are listed in S7 Table. We find that when 30% of the patches are masked, the pre-trained model performs best with the fine-tuning strategies.

S7 Table. The sensitivity results of mask rates on the SF dataset. The **Average** means the average of the simulations with $R_0 \in \{1.2, 1.8, 2.2, 2.8, 3.4\}$. For each R_0 and the **Average**, the best one is in **bold** and the runners-up is presented as underlined.

8 Analysis of the Patching Layer

In S4 Fig, we show attention maps from the Multi-head Attention, which include scenarios with and without patching (Patch vs. No Patch). These attention map is calculated by averaging the attention patterns $A(QW_Q^{(h)}, KW_K^{(h)})$ [5] over all the heads as described in the main body. The patching technique shortens the input length and consolidates attention scores across time steps, which results in more pronounced color variations in the attention maps compared to those without patching. This distinction highlights that attention weights between time steps are more defined and leads to the enhanced detection of crucial information at specific time steps. Further, the higher attention scores during the initial stages of outbreaks demonstrate their critical role in shaping the overall transmission dynamics, as indicated by the impact of initial R_0 on the transmission of the epidemic.

S4 Fig. Attention maps of the supervised Ct-Transformer with or without patching. (A) Attention map in stochastic simulation on the ER network with patching. (B) Attention map in stochastic simulation on the SF network with patching. (C) Attention map in stochastic simulation on the ER network without patching. (D) Attention map in stochastic simulation on the SF network without patching. All stochastic simulations with R_0=1.2.

We also investigate the impact of varying patch lengths on the performance of the Ct-Transformer. We adjust the patch lengths to $P = [2, 4, 6, 8, 10, 12, 14, 16, 18]$ and perform supervised training. The MAE losses with different patch lengths are shown in S5 Fig. The findings suggest that the Ct-Transformer benefits from longer patch lengths for the more accurate estimates of R_t and enhances computational efficiency. The optimal patch length varies between 4 and 8, depending on the specific dataset. For the ER dataset, the ideal patch length is 4, while for the SF synthetic dataset, it extends to 8.

S5 Fig. The MAE loss with varying patch lengths $P = [2, 4, 6, 8, 10, 12, 14, 16, 18]$ on the ER and SF datasets.

References

1. Perez L, Dragicevic S. An agent-based approach for modeling dynamics of contagious disease spread. International journal of health geographics. 2009;8(1):1–17. doi:10.1186/1476-072X-8-50.
2. Hay JA, Kennedy-Shaffer L, Kanjilal S, Lennon NJ, Gabriel SB, Lipsitch M, et al. Estimating epidemiologic dynamics from cross-sectional viral load distributions. Science. 2021;373(6552):eabh0635. doi:10.1126/science.abh0635.
3. Anderson RM, May RM. Infectious diseases of humans: dynamics and control. Oxford university press; 1991.
4. Wen R, Torkkola K, Narayanaswamy B, Madeka D. A multi-horizon quantile recurrent forecaster. arXiv preprint arXiv:171111053. 2017;doi:10.48550/arXiv.1711.11053.
5. Vaswani A, Shazeer N, Parmar N, Uszkoreit J, Jones L, Gomez AN, et al. Attention is all you need. Advances in neural information processing systems. 2017;30.
