# Supplementary material for: Estimating the time-varying effective reproduction number via Cycle Threshold-based Transformer
Source: PLoS Comput Biol. 2024 Dec 23;20(12):e1012694. doi: 10.1371/journal.pcbi.1012694 (PMC11706484; doi:10.1371/journal.pcbi.1012694)
Supplement: S1 Table — (PDF) [file pcbi.1012694.s007.pdf]

S1 Table. Parameters of the Agent-based SEIR Transmission Model and Ct Value Model.

Parameter	Description	Value
	Agent-based SEIR Transmission Model	
R_0	Basic reproduction number	Selected
$\langle k \rangle$	Average degree of contact network	10
N_{seed}	Number of initial infected seeds	1
N_{total}	Total number of individuals	100000
ε	Incubation period	$Poisson(\sigma), \sigma = 5.0$
ω	Infectious period	$Poisson(\gamma), \gamma = 4.0$
β	Probability of infection	$\beta = R_0 \times \frac{\gamma}{\langle k \rangle}$
	Ct Value Model	
$t_{eclipse}$	Time from infection to initial viral growth	0
C_{zero}	Ct value at time of infection	40.0
t_{peak}	Time from initial viral growth to peak viral load	$[Poisson(\sigma)]$
C_{peak}	Ct value at peak viral load	$Normal(22.3, 4.2)$
t_{end}	Time from peak viral load to recovery	$Normal(10.9, 0.94)$
