# Supplementary material for: Estimating the time-varying effective reproduction number via Cycle Threshold-based Transformer
Source: PLoS Comput Biol. 2024 Dec 23;20(12):e1012694. doi: 10.1371/journal.pcbi.1012694 (PMC11706484; doi:10.1371/journal.pcbi.1012694)
Supplement: S2 Table — (PDF) [file pcbi.1012694.s008.pdf]

S2 Table. Intervals d and the set D in the distribution of Ct values.

Inrervals d	Set D
3	$[16, 23], (23, 31], (31, 40]$
4	$[16, 22], (22, 28], (28, 34], (34, 40]$
5	$[16, 20], (20, 25], (25, 30], (30, 35], (35, 40]$
6	$[16, 20], (20, 24], (24, 28], (28, 32], (32, 36], (36, 40]$
8	$[16, 19], (19, 22], (22, 25], (25, 28], (28, 31], (31, 34], (34, 37], (37, 40]$
12	$[16, 18], (18, 20], (20, 22], (22, 24], (24, 26], (26, 28],$<br>$(28, 30], (30, 32], (32, 34], (34, 36], (36, 38], (38, 40]$
