# Supplementary material for: Estimating the time-varying effective reproduction number via Cycle Threshold-based Transformer
Source: PLoS Comput Biol. 2024 Dec 23;20(12):e1012694. doi: 10.1371/journal.pcbi.1012694 (PMC11706484; doi:10.1371/journal.pcbi.1012694)
Supplement: S3 Table — andThe Average means the average of simulations with R0 ∈ {1.2, 1.8, 2.2, 2.8, 3.4} in the testing set. For each R0 and the Average, the best results are in bold the runners-up are presented as underlined. (PDF) [file pcbi.1012694.s009.pdf]

S3 Table. The sensitivity results of intervals d on ER and SF datasets. The **Average** means the average of simulations with $R_0 \in \{1.2, 1.8, 2.2, 2.8, 3.4\}$ in the testing set. For each R_0 and the **Average**, the best results are in **bold** and the runners-up are presented as underlined.

d \ R_0		R_0=1.2	R_0=1.8	R_0=2.2	R_0=2.8	R_0=3.4	**Average**
ER dataset							
3	MAE	0.082	0.063	0.061	0.044	0.070	0.064
	RMSE	0.141	0.111	0.101	0.075	0.106	0.107
	R^2	0.813	0.971	0.985	0.995	0.993	0.951
4	MAE	0.084	**0.060**	**0.057**	**0.041**	0.075	**0.063**
	RMSE	0.142	**0.104**	**0.095**	**0.063**	0.118	**0.104**
	R^2	0.810	**0.975**	**0.988**	**0.997**	0.992	**0.952**
5	MAE	0.084	0.066	0.063	0.044	0.069	0.065
	RMSE	0.142	0.118	0.109	0.075	**0.097**	0.108
	R^2	0.806	0.967	0.982	0.995	**0.994**	0.949
6	MAE	0.085	0.066	0.064	0.042	**0.065**	0.064
	RMSE	0.147	0.118	0.108	0.069	0.098	0.108
	R^2	0.791	0.967	0.983	0.995	**0.994**	0.946
8	MAE	0.085	0.064	0.068	0.049	0.067	0.067
	RMSE	0.144	0.113	0.111	0.084	0.098	0.110
	R^2	0.800	0.970	0.981	0.992	**0.994**	0.947
12	MAE	**0.081**	0.063	0.061	0.050	0.088	0.070
	RMSE	**0.140**	0.109	0.102	0.078	0.133	0.112
	R^2	**0.816**	0.973	0.984	0.994	0.990	0.951
SF dataset							
3	MAE	0.103	0.114	0.082	0.092	0.110	0.100
	RMSE	0.188	0.255	0.187	0.217	0.211	0.212
	R^2	0.952	0.954	0.982	0.980	0.987	0.971
4	MAE	**0.094**	0.101	0.078	0.088	0.092	0.091
	RMSE	**0.161**	0.209	0.175	0.203	0.174	0.185
	R^2	**0.967**	0.965	**0.984**	0.983	0.990	**0.978**
5	MAE	0.100	**0.098**	0.078	0.087	**0.087**	**0.090**
	RMSE	0.173	**0.207**	0.175	0.202	**0.159**	**0.183**
	R^2	0.961	**0.966**	**0.984**	0.983	**0.991**	0.977
6	MAE	0.095	0.102	**0.076**	0.089	0.091	0.091
	RMSE	0.168	0.221	**0.174**	0.210	0.180	0.091
	R^2	0.963	0.963	**0.984**	0.981	0.989	0.976
8	MAE	0.109	0.105	0.085	0.091	0.098	0.098
	RMSE	0.199	0.225	0.191	0.210	0.187	0.202
	R^2	0.948	0.963	0.982	0.982	0.989	0.973
12	MAE	0.098	0.102	0.081	**0.083**	0.097	0.092
	RMSE	0.177	0.215	0.187	**0.196**	0.193	0.194
	R^2	0.958	0.964	0.982	**0.984**	0.989	0.975
