# Supplementary material for: Estimating the time-varying effective reproduction number via Cycle Threshold-based Transformer
Source: PLoS Comput Biol. 2024 Dec 23;20(12):e1012694. doi: 10.1371/journal.pcbi.1012694 (PMC11706484; doi:10.1371/journal.pcbi.1012694)
Supplement: S4 Table — The Average means the average of the simulations with R0 ∈ {1.2, 1.8, 2.2, 2.8, 3.4} in the testing set. For each R0 and the Average, the best results are in bold and the runners-up are presented as underlined. (PDF) [file pcbi.1012694.s010.pdf]

S4 Table. The sensitivity results of Ct variables (Var) on ER and SF datasets. The **Average** means the average of the simulations with $R_0 \in \{1.2, 1.8, 2.2, 2.8, 3.4\}$ in the testing set. For each R_0 and the **Average**, the best results are in **bold** and the runners-up are presented as underlined.

Var \ R_0		R_0=1.2	R_0=1.8	R_0=2.2	R_0=2.8	R_0=3.4	**Average**
ER dataset							
V1	MAE	0.088	0.064	0.063	0.046	0.066	0.065
	RMSE	0.152	0.114	0.106	0.077	0.097	0.109
	R^2	0.766	0.970	0.983	0.914	0.994	0.925
V2	MAE	0.086	0.061	0.059	0.046	0.077	0.066
	RMSE	0.144	0.109	0.101	0.075	0.113	0.108
	R^2	0.798	0.973	0.985	0.995	0.993	0.949
V3	MAE	0.084	**0.060**	**0.057**	**0.041**	0.075	0.063
	RMSE	**0.142**	**0.104**	**0.095**	**0.063**	0.118	0.104
	R^2	**0.810**	**0.975**	**0.988**	**0.997**	0.992	**0.952**
V4	MAE	**0.083**	**0.060**	0.058	0.044	**0.063**	**0.062**
	RMSE	0.144	0.106	0.098	0.073	**0.093**	**0.103**
	R^2	0.801	0.974	0.987	0.995	**0.995**	0.950
V5	MAE	0.085	0.064	0.062	0.045	0.070	0.065
	RMSE	0.148	0.110	0.106	0.075	0.103	0.108
	R^2	0.783	0.971	0.983	0.995	0.994	0.945
SF dataset							
V1	MAE	0.104	0.110	0.085	0.097	0.108	0.101
	RMSE	0.192	0.232	0.195	0.225	0.207	0.210
	R^2	0.949	0.960	0.981	0.981	0.987	0.972
V2	MAE	0.106	0.110	0.088	0.097	0.105	0.101
	RMSE	0.194	0.236	0.199	0.222	0.187	0.208
	R^2	0.950	0.959	0.980	0.980	0.989	0.972
V3	MAE	0.100	0.098	**0.078**	0.087	0.087	0.090
	RMSE	0.173	0.207	**0.175**	0.202	**0.159**	0.183
	R^2	0.961	0.966	**0.984**	0.983	**0.991**	0.977
V4	MAE	**0.096**	**0.095**	**0.078**	**0.085**	**0.086**	**0.088**
	RMSE	**0.167**	**0.195**	0.177	**0.198**	0.164	**0.180**
	R^2	**0.964**	**0.969**	0.983	**0.984**	0.990	**0.978**
V5	MAE	0.107	0.104	0.082	0.096	0.102	0.098
	RMSE	0.192	0.220	0.179	0.224	0.191	0.201
	R^2	0.952	0.964	**0.984**	0.980	0.989	0.974

V1: only the average of Ct values; **V2:** Average and skewness of Ct values; **V3:** only the distribution of Ct values; **V4:** Average and distribution of Ct values; **V5:** Average, skewness and distribution of Ct values.
