# Supplementary material for: Estimating the time-varying effective reproduction number via Cycle Threshold-based Transformer
Source: PLoS Comput Biol. 2024 Dec 23;20(12):e1012694. doi: 10.1371/journal.pcbi.1012694 (PMC11706484; doi:10.1371/journal.pcbi.1012694)
Supplement: S5 Table — (PDF) [file pcbi.1012694.s011.pdf]

S5 Table. Hyperparameters, tuning spaces, and the best hyperparameter settings for deep learning methods (Ct-Transformer, TFT, Transformer, and MLP) on the ER dataset and SF dataset.

Methods	Hyperparameter	Tuning Space	Best for ER dataset	Best for SF dataset
Ct-Transformer	d_{model}	256,512	512	512
	h_m	2,4,6,8,10	4	4
	N_{attn}	1,2,3,4,5,6	3	2
	d_{ff}	1024,2048,4096	2048	1024
	p_{drop}	0.1,0.2,0.3,0.4	0.1	0.1
	lr	5e-4,5e-5,5e-6	5e-4	5e-5
	N_{batch}	16,32,64,128	16	16
TFT	d_{model}	256,512	512	512
	h_m	2,4,6,8,10	8	6
	N_{attn}	1,2,3,4,5,6	2	2
	p_{drop}	0.1,0.2,0.3,0.4	0.1	0.1
	lr	5e-4,5e-5,5e-6	5e-5	5e-5
	N_{batch}	16,32,64,128	16	16
Transformer	d_{model}	256,512	512	512
	h_m	2,4,6,8,10	6	6
	N_{attn}	1,2,3,4,5,6	3	3
	d_{ff}	1024,2048,4096	2048	2048
	p_{drop}	0.1,0.2,0.3,0.4	0.1	0.1
	lr	5e-4,5e-5,5e-6	5e-4	5e-5
	N_{batch}	16,32,64,128	16	16
MLP	d_{model}	256,512	512	512
	d_{model_2}	1024,2048,4096	2048	2048
	N_{layers}	1,2,3,4,5,6	3	3
	p_{drop}	0.1,0.2,0.3,0.4	0.1	0.1
	lr	5e-4,5e-5,5e-6	5e-5	5e-5
	N_{batch}	16,32,64,128	32	32

$\mathbf{d_{model}}$: hidden size in the Ct-Transformer, TFT, Transformer, and MLP (the first layer);
$\mathbf{d_{model_2}}$: hidden size of layers (except the first layer) in the MLP;
$\mathbf{h_m}$: number of heads in the Ct-Transformer, TFT, and Transformer;
$\mathbf{N_{attn}}$: number of multi-attention layers in the Ct-Transformer, TFT, and Transformer;
$\mathbf{N_{layers}}$: number of linear layers in the MLP;
$\mathbf{d_{ff}}$: hidden size of the second linear layer in the feedforward network in the Ct-Transformer and TFT;
$\mathbf{p_{drop}}$: dropout rate;
lr: learning rate;
$\mathbf{N_{batch}}$: batch size.
