# Supplementary material for: Estimating the time-varying effective reproduction number via Cycle Threshold-based Transformer
Source: PLoS Comput Biol. 2024 Dec 23;20(12):e1012694. doi: 10.1371/journal.pcbi.1012694 (PMC11706484; doi:10.1371/journal.pcbi.1012694)
Supplement: S6 Table — For each detection scenario, the better one is presented as in bold. (PDF) [file pcbi.1012694.s012.pdf]

S6 Table. Results of the supervised Ct-Transformer and the EpiEstim method on the SF dataset under different detection scenarios. For each detection scenario, the better one is presented as in **bold**.

Test R_0		R_0=1.2		R_0=2.2		R_0=3.4	
Dection \ Method		Ct-Transformer	EpiEstim	Ct-Transformer	EpiEstim	Ct-Transformer	EpiEstim
Full Detection	MAE	**0.096**	0.120	**0.078**	0.121	**0.086**	0.208
	RMSE	**0.167**	0.216	**0.177**	0.196	**0.164**	0.387
	R^2	**0.964**	0.932	**0.984**	**0.984**	**0.990**	0.958
Scenario 1	MAE	**0.118**	0.182	**0.087**	0.183	**0.095**	0.315
	RMSE	**0.209**	0.381	**0.185**	0.345	**0.183**	0.682
	R^2	**0.938**	0.901	**0.982**	0.951	**0.989**	0.926
Scenario 2	MAE	**0.174**	0.214	**0.114**	0.215	**0.112**	0.370
	RMSE	**0.315**	0.485	**0.248**	0.440	**0.202**	0.869
	R^2	0.851	**0.872**	**0.964**	0.921	**0.986**	0.897
Scenario 3	MAE	**0.109**	0.173	**0.082**	0.174	**0.093**	0.299
	RMSE	**0.197**	0.375	**0.182**	0.341	**0.171**	0.672
	R^2	**0.947**	0.903	**0.982**	0.953	**0.989**	0.928
Scenario 4	MAE	**0.121**	0.195	**0.087**	0.197	**0.096**	0.338
	RMSE	**0.218**	0.457	**0.191**	0.414	**0.177**	0.818
	R^2	**0.933**	0.881	**0.980**	0.930	**0.989**	0.905
