# Supplementary material for: Estimating the time-varying effective reproduction number via Cycle Threshold-based Transformer
Source: PLoS Comput Biol. 2024 Dec 23;20(12):e1012694. doi: 10.1371/journal.pcbi.1012694 (PMC11706484; doi:10.1371/journal.pcbi.1012694)
Supplement: S7 Table — The Average means the average of the simulations with R0 ∈ {1.2, 1.8, 2.2, 2.8, 3.4}. For each R0 and the Average, the best one is in bold and the runners-up is presented as underlined. (PDF) [file pcbi.1012694.s013.pdf]

S7 Table. The sensitivity results of mask rates on the SF dataset. The **Average** means the average of the simulations with $R_0 \in \{1.2, 1.8, 2.2, 2.8, 3.4\}$. For each R_0 and the **Average**, the best one is in **bold** and the runners-up is presented as underlined.

Rate \ R_0		R_0=1.2	R_0=1.8	R_0=2.2	R_0=2.8	R_0=3.4	**Average**
0.1	MAE	0.095	0.099	0.088	0.103	0.081	0.093
	RMSE	**0.166**	0.204	0.201	0.247	0.153	0.194
	R^2	**0.965**	0.967	0.975	0.971	0.992	0.974
0.2	MAE	0.098	**0.087**	**0.075**	0.098	0.082	0.088
	RMSE	0.180	**0.179**	**0.171**	0.240	0.164	0.187
	R^2	0.934	**0.977**	0.983	0.972	0.989	0.971
0.3	MAE	**0.094**	0.094	0.081	**0.082**	**0.074**	**0.085**
	RMSE	0.170	0.188	0.175	**0.183**	**0.144**	**0.172**
	R^2	0.958	0.973	**0.984**	**0.986**	**0.994**	**0.979**
0.4	MAE	0.105	0.101	0.092	0.097	0.092	0.097
	RMSE	0.191	0.214	0.213	0.224	0.178	0.204
	R^2	0.930	0.955	0.970	0.976	0.988	0.964
