# Supplementary material for: Estimating the time-varying effective reproduction number via Cycle Threshold-based Transformer
Source: PLoS Comput Biol. 2024 Dec 23;20(12):e1012694. doi: 10.1371/journal.pcbi.1012694 (PMC11706484; doi:10.1371/journal.pcbi.1012694)
Supplement: Attachment — Submitted filename: review_rt.pdf [file pcbi.1012694.s014.pdf]

Review on
Estimating the time-varying effective reproduction number via Cycle Threshold-based Transformer

The paper proposed an accurate, attention-based deep neural network method, called Ct-Transformer, to estimate time-varying effective reproduction number (Rt) by transforming time-varying cycle threshold (Ct) values. Compared to many existing methods, the proposed method has fewer assumptions on the knowledge of epidemics, such as generation time distribution, which is hard to reach at the early stage of epidemics. It does not use incidence observations as input, so it is not affected by the bias in incidence due to, such as, reporting delays. Meaningful visualizations are generated to help with a better illustration. A more thorough discussion on methodology and experiments and a better explanation on the scope of contributions can largely enrich the paper. Clarification on parameters, math notations and abbreviations can make the work clearer to understand. Some related existing work on effective reproduction number estimation is recommended to include.

Major comments

The paper can benefit from a clarification of contributions and a more thorough discussion on experimental settings. A more detailed explanation on the synthetic data generation procedure can be useful for the readers to understand the proposed method.

1. The proposed Ct-Transformer model is designed to estimate time-varying effective reproduction numbers, while effective reproduction number can be defined in two ways—instantaneous reproduction number and case reproduction number Gostic et al. (2020). Does the proposed model focus on one of them or is it able to estimate both? It's worth clarifying the scope of contribution at the beginning and, if necessary, illustrating both types of effective reproduction number estimates.

2. The authors used two (ER and SF) contact networks with different values of R_0 in simulation. The networks help with representing the spread of epidemic among population following different contact patterns. What are the main differences between the two networks in terms of the contact patterns they can generate that make it worthwhile considering both rather than one of them? The authors also mentioned "degree" for the networks in line 87, but did not interpret it. An interpretation on what the "degree" level means to the epidemic transmission using the networks can be helpful

to clarify the confusion. When R_0 is first mentioned in line 87, it's not defined. Does R_0 represent R_t at time $t = 0$ or, by convention, the basic reproduction number?

3. The authors provided a thorough discussion on the parameters in the proposed method, while the parameters used in the alternatives in the experiments are not completely listed. A complete discussion on the choice of parameters in the alternative methods in the experiments can make the experimental settings more complete and choosing the parameters to maximize the performance of alternative methods can make the experimental results more plausible.

4. In experimental results in Fig 3 in the manuscript and Figure `S2_Fig` in the Appendix, the R_t estimates start from (around) 20 days since start of outbreak. An explanation on why the first few days, 20 days specifically, are excluded from estimation or visualization can be useful for a better understanding and make the section more self-contained. Consequently, interpretation (and math notations, if necessary) on whether the accuracy of estimation is measured only after 20 days since start of outbreak can be helpful as well. Additionally, a brief explanation in the figure captions can make the figures more self-contained.

Minor comments

A more thorough literature review can make the paper more self-contained. Readers can also benefit from a more detailed explanation on parameters and clarification of a few abbreviations. Details are listed as follows.

1. In line 66, the authors stated their method outperforms other alternatives. A brief discussion on the specific aspects, e.g., accuracy of Rt estimation, that the proposed method improves upon compared to others can make the point more clearly stated.

2. The literature review in lines 11–21 can be enhanced by citing relevant literatures on accurate time-varying effective reproduction estimation using generation time distribution, e.g., Abbott et al. (2020); Gressani et al. (2022); Liu et al. (2023); Parag (2021), which are alternatives to EpiEstim.

3. The abbreviation "GNN" is not defined when it's first mentioned in line 52.

4. The abbreviation "LSTM" is not defined when it's first mentioned in line 54.

5. In line 105, Ct value at each time is defined as a vector of dimension M. What does the dimension refer to? Is it a hyperparameter of users' choice?

6. The abbreviation "RNN" is not defined when it's first mentioned in line 135.

References

Abbott, S., Hellewell, J., Thompson, R. N., Sherratt, K., Gibbs, H. P., Bosse, N. I., Munday, J. D., Meakin, S., Doughty, E. L., Chun, J. Y. et al. (2020) Estimating the time-varying reproduction number of SARS-CoV-2 using national and subnational case counts. *Wellcome Open Research*, **5**, 112. https://doi.org/10.12688/wellcomeopenres.16006.2.

Gostic, K. M., McGough, L., Baskerville, E. B., Abbott, S., Joshi, K., Tedijanto, C., Kahn, R., Niehus, R., Hay, J. A., De Salazar, P. M. et al. (2020) Practical considerations for measuring the effective reproductive number, R_t. *PLoS Computational Biology*, **16**, e1008409. https://doi.org/10.1371/journal.pcbi.1008409.

Gressani, O., Wallinga, J., Althaus, C. L., Hens, N. and Faes, C. (2022) Epilps: A fast and flexible Bayesian tool for estimation of the time-varying reproduction number. *PLoS Computational Biology*, **18**, e1010618. https://doi.org/10.1371/journal.pcbi.1010618.

Liu, J., Cai, Z., Gustafson, P. and McDonald, D. J. (2023) Rtestim: Effective reproduction number estimation with trend filtering. *medRxiv*. URL: https://www.medrxiv.org/content/early/2023/12/18/2023.12.18.23299302.

Parag, K. V. (2021) Improved estimation of time-varying reproduction numbers at low case incidence and between epidemic waves. *PLoS Computational Biology*, **17**, e1009347. https://doi.org/10.1371/journal.pcbi.1009347.
