# Supplementary material for: Estimating the time-varying effective reproduction number via Cycle Threshold-based Transformer
Source: PLoS Comput Biol. 2024 Dec 23;20(12):e1012694. doi: 10.1371/journal.pcbi.1012694 (PMC11706484; doi:10.1371/journal.pcbi.1012694)
Supplement: Attachment — Submitted filename: review_comments.docx [file pcbi.1012694.s015.docx]

**Estimating the time-varying effective reproduction number via Cycle Threshold-based**

**Transformer**

This paper discusses the use of Ct values to estimate the effective reproduction number. In particular, the authors propose a new method called the Cycle Threshold-based Transformer (Ct-Transformer) for estimating the time-varying effective reproduction number (Rt) of infectious diseases. The authors discuss limitations of existing methods, the advantages of the Ct-Transformer, and its performance compared to other methods using synthetic and real-world datasets. They discuss relevant literature and discusses the drawbacks in the current literature and relevant packages such as ViroSolver and EpiEstim whilst highlighting the novelty of the work. It also presents the results of supervised and self-supervised learning of the Ct-Transformer, as well as the impact of detection rates on its performance. The manuscript includes details on the architecture, training process, experimental settings, and evaluation metrics used in the study. The results compare the authors’ methods with other methods in the literature. However, I believe the paper will benefit with some improvements, particularly around the structure and the target audience.

I have some minor comments that are listed below.

**Minor comments:**

1. In the last two paragraphs of the introduction, it is not immediately clear what Transformers are. It would be great if the authors can explicitly briefly explain what they are before the last paragraph.
2. It would be helpful if the authors can include some details about the applicability of this work with some examples in the introduction to clearly state and highlight the novelty and objectives of this work.
3. It will be helpful to first include the section on the theory related to the Ct transformers rather than Data to draw the readers’ attention as to why those specific data were generated/ chosen and to connect the theory with application to data. Also consider adding visualisations of both synthetic and real-world data in these sections.
4. Please consider adding more details about the real dataset in the data section.
5. Given that synthetic data are dependent on different contact networks (ER and SF), it would be helpful to explain briefly what they are for the benefit of non-specialised audience.
6. Authors mention that methods like ViroSolver needs prior knowledge as it uses Bayesian inference. On the other hand, the Ct Transformer also requires prior knowledge about the Ct evolution especially for a newly emerging epidemic. It would be great if the authors can explain how recalibration of the Ct Transformer can be used, particularly to new outbreaks or different countries with distinct contact patterns.
7. The paper suggests required knowledge in machine learning and related methods apart from infectious disease related theory. If the authors already haven’t done so, it may be helpful for the non-specialised audience to access codes and run a reproducible example using the data used.
8. Further to point 6 above, perhaps it would be great if the authors can consider the target audience (i.e., who will benefit from this work more?) and tailor the language accordingly. Similarly consider providing more details and explanations on the machine learning concepts and methods.
9. There are a lot of acronyms that the reader may find hard to keep up and some of them are not clearly explained either. It would be great if the authors can make sure all the abbreviations and acronyms are clearly explained throughout the manuscript.
