# Supplementary material for: Estimating the time-varying effective reproduction number via Cycle Threshold-based Transformer
Source: PLoS Comput Biol. 2024 Dec 23;20(12):e1012694. doi: 10.1371/journal.pcbi.1012694 (PMC11706484; doi:10.1371/journal.pcbi.1012694)
Supplement: Attachment — Submitted filename: response_letter.docx [file pcbi.1012694.s016.docx]

Dear Editor,

Please find enclosed the revised version of our manuscript “Estimating the time-varying effective reproduction number via Cycle Threshold-based Transformer” (Tracking#: PCOMPBIOL-D-24-00718) by Zhang et al.

We would like to thank you for giving us the opportunity to revise our manuscript. We are also truly grateful to the reviewers for their thorough assessment of our manuscript that was instrumental to greatly improving our work.

We enclose a point-by-point response to all comments and remarks of the reviewers, explaining the corresponding changes made in the manuscript.

We believe the manuscript has now considerably improved, and we dare to hope that it is now suitable for publication in PLoS Computational Biology.

Sincerely yours,

Gui-Quan Sun and Quan-Hui Liu

**Detailed reply to Reviewers**

We are extremely grateful to the editor and the two reviewers for their thorough assessment of the manuscript. Their remarks and comments truly helped us to considerably improve our manuscript.

Reviewer #1:

This paper discusses the use of Ct values to estimate the effective reproduction number. In particular, the authors propose a new method called the Cycle Threshold-based Transformer (Ct-Transformer) for estimating the time-varying effective reproduction number (Rt) of infectious diseases. The authors discuss limitations of existing methods, the advantages of the Ct-Transformer, and its performance compared to other methods using synthetic and real-world datasets. They discuss relevant literature and discusses the drawbacks in the current literature and relevant packages such as ViroSolver and EpiEstim whilst highlighting the novelty of the work. It also presents the results of supervised and self-supervised learning of the Ct-Transformer, as well as the impact of detection rates on its performance. The manuscript includes details on the architecture, training process, experimental settings, and evaluation metrics used in the study. The results compare the authors’ methods with other methods in the literature. However, I believe the paper will benefit with some improvements, particularly around the structure and the target audience.

We would like to thank the reviewer for taking the time to review our manuscript and for the constructive comments. We are glad to hear that the reviewer thinks that our paper “will benefit with some improvements, particularly around the structure and the target audience”.

I have some minor comments that are listed below.

Minor

1.1 In the last two paragraphs of the introduction, it is not immediately clear what Transformers are. It would be great if the authors can explicitly briefly explain what they are before the last paragraph.

Thank you for this suggestion. We have added the introductions about the deep learning and a detailed description of Transformers prior to discussing their application (**Line: 50**):

“*With the increasing availability of data and powerful learning ability, the deep learning as a nonlinear mathematical method is widely used in image recognitions [35], natural language processing [36], and time series forecasting [37], etc. The core of deep learning is to build machine learning architecture model with multiple hidden layers, to train them on large-scale data, and obtain a large amount of more representative feature information [38]. And then, some samples can be used to test the model and finally improve the model performance such as the prediction accuracy. The early notable deep learning models include Recurrent Neural Networks (RNNs) [39] and Long Short-Term Memory (LSTM) networks [39]. Therein, the architecture of RNNs is designed to extract temporal feature from sequence data, while the LSTM, as an advanced form of RNNs, is built for capturing long-term dependencies in sequence data. During the COVID-19 period, the deep learning models have been applied in the field of epidemiology [40-43]. Davahli et al. [40] develop two types of Graph Neural Networks (GNN) models [44], which are designed to handle graph-structured data, to estimate R_t_ using COVID-19 cases from 22 January 2020 to 26 November 2020 in the United States. Gatto et al. [41] utilize LSTM architecture to predict the daily trend of time-varying reproduction number (R_t_). They find the neural network-based models accurately predict R_t_ in the region and autonomous province of Italy when sufficient epidemiological data is available. Although the above architectures of neural network are effective in time series prediction tasks, they still exhibit the vanishing gradients and hard to capture long-range dependencies. A significant advancement in modern machine learning is that Vaswani et al. [45] propose the Transformer, an encoder-decoder based neural network architecture with the novel self-attention mechanism. The encoder is designed to process the input sequence, and the decoder generates output sequences, and both of them consist multiple layers and communicating via attention mechanism. The self-attention mechanism allows the model to weigh the importance of different parts of the input sequence and enables it to capture the long-range dependencies efficiently. Both the computation efficiency and its ability to handle with long-range dependencies have made the Transformer architecture a mainstream choice in deep learning models.*”

1.2 It would be helpful if the authors can include some details about the applicability of this work with some examples in the introduction to clearly state and highlight the novelty and objectives of this work

Thank you for this suggestion. We have revised the last paragraph of **Introduction** and highlighted the novelty and objectives of this work (**Line: 75**):

“*The main aim of this study is to estimate the case reproduction number from the time-varying distribution of Ct values based on deep learning models. In order to better capture the long-range dependencies and extract the temporal features, we propose the Cycle Threshold-based Transformer (i.e., Ct-Transformer) with the canonical components, including the patching layer, the gated recurrent unit network and the multi-head attention layer. The detailed descriptions of the above components in the architecture of Ct-Transformer are presented in Materials and Methods. To be noted that, both the supervised learning and the self-supervised learning of Ct-Transformer are developed. Therein, the supervised learning of Ct-Transformer is trained on a large number of datasets with labels, while the self-supervised one can be trained on the datasets without labels and fine-turning the pre-trained model on small portion of labeled data, which finally both of them can be used to estimate R_t_. By evaluating the Ct-Transformer on the synthetic datasets, we find the supervised learning of Ct-Transformer performs best in all Ct-based deep learning and statistical methods. More in detail, the supervised learning of Ct-Transformer achieves a 31.1% reduction in MAE and a 27.0% reduction in RMSE, compared with other cycle thresholds based deep learning methods. We also find the proposed Ct-Transformer is robust to both the limited and the time-varying detection resources. It achieves similar performance or better performance in the situations with 25% detection rate compared with the traditional incidence-based R_t_ estimation method in the situations with 100% detection rate. Lastly, we pre-train the self-supervised learning of Ct-Transformer on synthetic datasets, and then fine-turn the pre-trained model on the Hong-Kong COVID-19 data. We obtain a 24% reduction in MAE compared with the result reported in [31].*

*In this study, we propose the Cycle Threshold-based Transformer (Ct-Transformer), which is a deep learning-based method to estimate R_t_. The self-supervised learning of Ct-Transformer can be used to estimate R_t_ of the same disease on other regions or countries by fine-turning on small portion of real labelled data. The proposed Ct-Transformer is evaluated on both the synthetic and real datasets, which proves to be effective and robust to the detection resources. The ablation experiments also show the necessity of each canonical components in the design on the architecture of Ct-Transformer. In summary, this study offers public health authorities a deep learning method for estimating R_t_ without the time delays inherent in traditional incidence-based methods and few prior knowledge requirement. Additionally, it can be easily adapted to estimate R_t_ for newly emerging diseases by adjusting the model with small portion of real data.*”

1.3 It will be helpful to first include the section on the theory related to the Ct transformers rather than Data to draw the readers’ attention as to why those specific data were generated/ chosen and to connect the theory with application to data. Also consider adding visualizations of both synthetic and real-world data in these sections.

We apologize for the inappropriate structure of manuscript. We have took the suggestion of the reviewer and exchanged the order of the section about **the theory related to the Ct-Transformer** and **the Data** section to make the manuscript being easily understood in this revised manuscript.

We are sorry for the unclear description of both the synthetic and real-world data in **the** **Data** section. We have revised this section and the description of visualizations for both the synthetic and real-world data.

For better elaborating the generating procedure of the synthetic dataset, we have added a subsection about the **SEIR Transmission Model and the Ct Value Model** on the section of **Materials and methods**, which is presented as follows (**Line: 194**) :

**“*SEIR Transmission Model and the Ct Value Model***

*We adopt the agent-based stochastic Susceptible-Exposed-Infectious-Removed (SEIR) transmission model [57] and the Ct value model [25] to produce the synthetic dataset. The SEIR transmission model classifies the population into four types: susceptible (S), exposed (E), infectious (I) and removed (R). Therein, the susceptible state represents an individual who is not infected but can be infected, the exposed state represents an individual who is infected but not infectious, and the infectious state represents an individual who is infected and can infect susceptible individuals. Lastly, the removed state represents an individual who has recovered from the infectious state and can not be infected again. The flow of these states is presented in Fig 1A.*

*We employ the Ct value model reported in [25] to simulate the evolution of viral load (characterized by the Ct value) within host since the infection. In specific, we assume that the observed Ct value a days after infection, Ct(a), is represented by Eq. (S1) in S1 Supplementary Methods (1. Parameters of the Transmission Model). As shown in S1 Fig, the trajectories of Ct values capture the variation resulting from both swabbing variability and individual-level differences in viral kinetics, as shown in S1 Fig.*

*The parameters used to simulate both the SEIR model and the Ct value model are listed in S1 Table in S1 Supplementary Methods (1. Parameters of the Transmission Model).*”

We also have revised the steps to generate the synthetic data, which is listed as follows (**Line: 211**):

**“*Synthetic Datasets.*** *The synthetic dataset are generated with the same framework but with different transmission parameters. For each simulation, the data is produced with following three steps: 1) On each day of the simulation, we simulate the SEIR transmission model with one time step (i.e., one day) and produce the newly infected individuals at day t; 2) A subset of individuals are sampled on each day of the simulation determined by the detection scenarios described at the subsection of Impact of Detection Rates on Performance in Results. 3) For each individual i sampled on day a, a Ct value Ct(a-t) is generated according to the Ct value model. Therein, a-t represents the infection time of individual i, which is described in S1 Supplementary Methods (1. Parameters of the Transmission Model). To be noted that, at step 1), the micro-transmission chains including the time of each infected individual, and his/her infectees are kept, which were used to calculate the time-varying effective reproduction number R_t_. The details about the calculation are presented in S1 Supplementary Methods (2. Calculation of R_t_ based on Micro-transmission Chains).*”

We presented the visualization of the synthetic dataset and the related analysis result on the subsection of the **Relationship between Ct values and R_t_** in the section of **Results**. For your convenience, we presented both the figure and the related description of the figures in the follow.

“*As shown in Fig 2A, we find the ratio of the number of newly infected individuals to the currently infected individuals keeps high during the early stage of the epidemic. This ratio then decreases sharply as the epidemic reaches its peak, and finally keeps low towards the end of the epidemic. This trend implies that during the early stage of the epidemic, random sampling is more likely to detect individuals who have been recently infected and exhibit lower Ct values. Conversely, during the declining stage of the epidemic, the sampled infections present higher Ct values as they approach recovery. This is revealed in the average of Ct values in Fig 2C.*

*The temporal evolution of R_t_ and the average of Ct values are respectively presented in Fig 2B and Fig 2D. We find that R_t_ keeps relatively stable during the early stage of the epidemic and then undergoes a rapid decline when reaching the peak. This trend is revealed by the evolution of the average of Ct values, which initially remains steady but then experiences a sharp increase as the transmission of the epidemic. In the meanwhile, a lower average of Ct values at the onset of the epidemic is found in the simulated epidemic with a higher R_0_. These results imply the time-varying distribution of Ct values may serve as an observable proxy to infer the transmission dynamics of an epidemic [25].* “

The description of Real-world Datasets are updated in this revised version as follows (**Line : 242**):

“***Real-world Dataset.*** *In addition to the synthetic datasets, we also evaluate the performance of the Ct-Transformer on the Real-world dataset (i.e., Hong Kong COVID-19 dataset). The Hong Kong COVID-19 dataset includes two consecutive waves of the spread of the COVID-19 in Hong Kong, which are respectively as the third wave spanning July to August 2020 and the fourth wave from November 2020 to March 2021. During these two periods, 8646 COVID-19 cases are detected through both the clinical diagnosis and the public health surveillance. In the meanwhile, the first available records of Ct values (derived from RT-qPCR tests targeting E gene) for the detected COVID-19 cases are collected. We align the dates of first available record of Ct value of all detected cases and compute the mean and the skewness of Ct values at each date during the third and forth waves of the spread of COVID-19 in Hong Kong, as is shown in Fig 4A. Besides, the estimated time series of R_t_ reported in [31] are included in this Real-world dataset, presented as in Fig 4B. To test the performance of Ct-Transformer on this real-world dataset, we adjust the input format of the Ct-Transformer with the mean and the skewness of Ct values at each time step. We adopt the same method reported in [31] and divide the real-world dataset into the training, validation and testing sets, which are respectively as follows:*

*Training set, from Jul 6th 2020 to Sept 17th 2020.*

*Validation set, from Sept 18th 2020 to Nov 15th 2020.*

*Testing set, from Nov 16th 2020 to Mar 23rd 2021.*”

We presented the visualization of the Real-world dataset and the related analysis result on the subsection of the **Real-world Data Verification** in the section of **Results**. For your convenience, we presented both the figure and the related description of the figures in the follow (**Line: 376**):

“*As shown in Fig. 4A, the average of Ct values over time display a distinctly different trend compared to both the skewness of Ct values and the R_t_ trend, as illustrated in Fig. 4 B. This behavior mirrors the phenomena observed in the synthetic dataset, as reported in Figs. 2B and 2D.*”

1.4 Please consider adding more details about the real dataset in the data section

We apologize for the lack of clarity about the real-world dataset. We have added the details and revised the description about the real-world dataset in **the Data** section. Please see **the response of 1.3**.

1.5 Given that synthetic data are dependent on different contact networks (ER and SF), it would be helpful to explain briefly what they are for the benefit of non-specialised audience.

We apologize for the lack of clarity, and we are grateful to the reviewer for pointing out that we were missing the introduction of these two contact networks (ER and SF). In this revised version, we added the detailed introduction about both type of contact networks in the Data section (**Line: 223**):

“*We consider multiple scenarios by modeling outbreaks on two distinct types of contact networks: the Erdős-Rényi (ER) network [58] and Scale-Free (SF) network [59]. The ER network is a random graph where edges between nodes are formed independently and randomly, and is used as a theoretical baseline to study the spread of disease on networks. The degree of a node, i.e., the number of neighbors or edges connected to a node in the ER network, approaches a Poission distribution, which is relatively narrow and most of nodes in the ER network having similar degrees. Thus, the ER network is used to characterize the homogenous contact pattern of population. In contrast, the SF network is developed to characterize the degree distribution of real networks, which usually follows the power law. It means that a small number of nodes in the SF network have a very high number of connections, while most other nodes have relatively few connections. We set the average degree of both type of contact network equal to 10 and simulate the outbreaks with different value of R_0_ (the average number of secondary cases generated by a typical infectious individual over the entire course of the infectious period in a fully susceptible population) to represent the modeled epidemic with different transmission abilities.*”

1.6 Authors mention that methods like ViroSolver needs prior knowledge as it uses Bayesian inference. On the other hand, the Ct Transformer also requires prior knowledge about the Ct evolution especially for a newly emerging epidemic. It would be great if the authors can explain how recalibration of the Ct Transformer can be used, particularly to new outbreaks or different countries with distinct contact patterns.

We apologize for the lack of clarity. The ViroSolver needs the prior knowledge including both the transmission dynamic such as the prior distribution for the epidemic seed time, the constant exponential growth of infection incidence, etc., and the within-host virus kinetics characterized by the Ct value model. While the Ct-Transformer only needs the within-host virus kinetics. We have revised this part of **Discussion** as follows (**Line: 446**):

“*However, this method needs the prior knowledges including both the transmission dynamic such as the prior distribution for the epidemic seed time, the constant exponential growth of infection incidence, etc., and the within-host virus kinetics characterized by the Ct value model.*”

The total calibration process of self-supervised learning of Ct-Transformer consists of two steps.

- The first step is called the masked self-supervised training which outputs the pre-trained model. In specific, the self-supervised learning of Ct-Transformer is pretrained on a large synthetic datasets without labels, which are produced by simulating the outbreak with the same within-host virus kinetics as the epidemic that we are going to predict in other countries. To be noted that, we only require the same within-host virus kinetics when producing the synthetic datasets. The within-host virus kinetics is usually characterized by the cycle threshold value model, which can be obtained by fitting the cycle threshold values of detected cases with multiple tests, such as the RT-qPCR tests.
- The second step is supervised fine-tuning the pre-trained model. More in detail, we fine-turn the pre-trained Ct-Transformer in part of real data collected from the target country where the infectious disease of the same within-host virus kinetics is spreading. Thus, the model is calibrated, which can be used to estimate the Rt of the target country.

Moreover, if we find the same epidemic occurs in other country, we can fine-turn or recalibrate the pre-trained model on part of real data collected from this country, to make the model adapted to this new country.

The detailed procedures of calibrating the model are listed in Line: 175.

In addition, on the subsection of **the self-supervised learning results** in the section of **Results**, we pre-train the Ct-Transformer on the ER data, and fine-turn the pretrained model on both the SF data and the real-world data. The test results show the pre-trained model can be easily and efficiently adapted to the population with different contact patterns.

1.7 The paper suggests required knowledge in machine learning and related methods apart from infectious disease related theory. If the authors already haven’t done so, it may be helpful for the non-specialised audience to access codes and run a reproducible example using the data used.

Thank you for this nice suggestion. We have made the code, data and readme publicly available in https://github.com/xiaohuahua1/Ct-Transformer.

1.8 Further to point 6 above, perhaps it would be great if the authors can consider the target audience (i.e., who will benefit from this work more?) and tailor the language accordingly. Similarly consider providing more details and explanations on the machine learning concepts and methods.

Thank you for this suggestion. We have added more detailed introduction into the concepts of deep learning, the classical deep learning models and their limitations, to ensure that readers from various backgrounds can grasp the significance of our work and analysis at the end of the introduction (complete the last three paragraphs of **Introduction**):

“*With the increasing availability of data and powerful learning ability, the deep learning as a nonlinear mathematical method is widely used in image recognitions [35], natural language processing [36], and time series forecasting [37], etc. The core of deep learning is to build machine learning architecture model with multiple hidden layers, to train them on large-scale data, and obtain a large amount of more representative feature information [38]. And then, some samples can be used to test the model and finally improve the model performance such as the prediction accuracy. The early notable deep learning models include Recurrent Neural Networks (RNNs) [39] and Long Short-Term Memory (LSTM) networks [39]. Therein, the architecture of RNNs is designed to extract temporal feature from sequence data, while the LSTM, as an advanced form of RNNs, is built for capturing long-term dependencies in sequence data. During the COVID-19 period, the deep learning models have been applied in the field of epidemiology [40-43]. Davahli et al. [40] develop two types of Graph Neural Networks (GNN) models [44], which are designed to handle graph-structured data, to estimate R_t_ using COVID-19 cases from 22 January 2020 to 26 November 2020 in the United States. Gatto et al. [41] utilize LSTM architecture to predict the daily trend of time-varying reproduction number (R_t_). They find the neural network-based models accurately predict R_t_ in the region and autonomous province of Italy when sufficient epidemiological data is available. Although the above architectures of neural network are effective in time series prediction tasks, they still exhibit the vanishing gradients and hard to capture long-range dependencies. A significant advancement in modern machine learning is that Vaswani et al. [45] propose the Transformer, an encoder-decoder based neural network architecture with the novel self-attention mechanism. The encoder is designed to process the input sequence, and the decoder generates output sequences, and both of them consist multiple layers and communicating via attention mechanism. The self-attention mechanism allows the model to weigh the importance of different parts of the input sequence and enables it to capture the long-range dependencies efficiently. Both the computation efficiency and its ability to handle with long-range dependencies have made the Transformer architecture a mainstream choice in deep learning models.*

*The main aim of this study is to estimate the case reproduction number from the time-varying distribution of Ct values based on deep learning models. In order to better capture the long-range dependencies and extract the temporal features, we propose the Cycle Threshold-based Transformer (i.e., Ct-Transformer) with the canonical components, including the patching layer, the gated recurrent unit network and the multi-head attention layer. The detailed descriptions of the above components in the architecture of Ct-Transformer are presented in Materials and Methods. To be noted that, both the supervised learning and the self-supervised learning of Ct-Transformer are developed. Therein, the supervised learning of Ct-Transformer is trained on a large number of datasets with labels, while the self-supervised one can be trained on the datasets without labels and fine-turning the pre-trained model on small portion of labeled data, which finally both of them can be used to estimate R_t_. By evaluating the Ct-Transformer on the synthetic datasets, we find the supervised learning of Ct-Transformer performs best in all Ct-based deep learning and statistical methods. More in detail, the supervised learning of Ct-Transformer achieves a 31.1% reduction in MAE and a 27.0% reduction in RMSE, compared with other cycle thresholds based deep learning methods. We also find the proposed Ct-Transformer is robust to both the limited and the time-varying detection resources. It achieves similar performance or better performance in the situations with 25% detection rate compared with the traditional incidence-based R_t_ estimation method in the situations with 100% detection rate. Lastly, we pre-train the self-supervised learning of Ct-Transformer on synthetic datasets, and then fine-turn the pre-trained model on the Hong-Kong COVID-19 data. We obtain a 24% reduction in MAE compared with the result reported in [31].*

*In this study, we propose the Cycle Threshold-based Transformer (Ct-Transformer), which is a deep learning-based method to estimate R_t_. The self-supervised learning of Ct-Transformer can be used to estimate R_t_ of the same disease on other regions or countries by fine-turning on small portion of real labelled data. The proposed Ct-Transformer is evaluated on both the synthetic and real datasets, which proves to be effective and robust to the detection resources. The ablation experiments also show the necessity of each canonical components in the design on the architecture of Ct-Transformer. In summary, this study offers public health authorities a deep learning method for estimating R_t_ without the time delays inherent in traditional incidence-based methods and few prior knowledge requirement. Additionally, it can be easily adapted to estimate R_t_ for newly emerging diseases by adjusting the model with small portion of real data.*”

1.9 There are a lot of acronyms that the reader may find hard to keep up and some of them are not clearly explained either. It would be great if the authors can make sure all the abbreviations and acronyms are clearly explained throughout the manuscript.

We apologize for any confusion caused by the unclear acronyms in the manuscript. We have thoroughly reviewed and corrected them for better clarity.

Reviewer #2 (Remarks to the Author):

The paper proposed an accurate, attention-based deep neural network method, called Ct-Transformer, to estimate time-varying effective reproduction number (Rt) by transforming time-varying cycle threshold (Ct) values. Compared to many existing methods, the proposed method has fewer assumptions on the knowledge of epidemics, such as generation time distribution, which is hard to reach at the early stage of epidemics. It does not use incidence observations as input, so it is not affected by the bias in incidence due to, such as, reporting delays. Meaningful visualizations are generated to help with a better illustration. A more thorough discussion on methodology and experiments and a better explanation on the scope of contributions can largely enrich the paper. Clarification on parameters, math notations and abbreviations can make the work clearer to understand. Some related existing work on effective reproduction number estimation is recommended to include.

We would like to thank the reviewer for taking the time to review our manuscript and for the constructive comments. We have added the discussion on the methodology and experiment part. We also have revised the contribution of this work on the introduction. We have thoroughly reviewed and corrected the unclear acronyms for better clarity. Further, we have added some related existing works on effective reproduction number estimation in the Introduction.

Major comments

The paper can benefit from a clarification of contributions and a more thorough discussion on experimental settings. A more detailed explanation on the synthetic data generation procedure can be useful for the readers to understand the proposed method.

Thank you for this suggestion. As with **Response 1.2**, we have revised the Introduction to clarify the contribution of this work (**Line: 75**):

“*The main aim of this study is to estimate the case reproduction number from the time-varying distribution of Ct values based on deep learning models. In order to better capture the long-range dependencies and extract the temporal features, we propose the Cycle Threshold-based Transformer (i.e., Ct-Transformer) with the canonical components, including the patching layer, the gated recurrent unit network and the multi-head attention layer. The detailed descriptions of the above components in the architecture of Ct-Transformer are presented in Materials and Methods. To be noted that, both the supervised learning and the self-supervised learning of Ct-Transformer are developed. Therein, the supervised learning of Ct-Transformer is trained on a large number of datasets with labels, while the self-supervised one can be trained on the datasets without labels and fine-turning the pre-trained model on small portion of labeled data, which finally both of them can be used to estimate R_t_. By evaluating the Ct-Transformer on the synthetic datasets, we find the supervised learning of Ct-Transformer performs best in all Ct-based deep learning and statistical methods. More in detail, the supervised learning of Ct-Transformer achieves a 31.1% reduction in MAE and a 27.0% reduction in RMSE, compared with other cycle thresholds based deep learning methods. We also find the proposed Ct-Transformer is robust to both the limited and the time-varying detection resources. It achieves similar performance or better performance in the situations with 25% detection rate compared with the traditional incidence-based R_t_ estimation method in the situations with 100% detection rate. Lastly, we pre-train the self-supervised learning of Ct-Transformer on synthetic datasets, and then fine-turn the pre-trained model on the Hong-Kong COVID-19 data. We obtain a 24% reduction in MAE compared with the result reported in [31].*

*In this study, we propose the Cycle Threshold-based Transformer (Ct-Transformer), which is a deep learning-based method to estimate R_t_. The self-supervised learning of Ct-Transformer can be used to estimate R_t_ of the same disease on other regions or countries by fine-turning on small portion of real labelled data. The proposed Ct-Transformer is evaluated on both the synthetic and real datasets, which proves to be effective and robust to the detection resources. The ablation experiments also show the necessity of each canonical components in the design on the architecture of Ct-Transformer. In summary, this study offers public health authorities a deep learning method for estimating R_t_ without the time delays inherent in traditional incidence-based methods and few prior knowledge requirement. Additionally, it can be easily adapted to estimate R_t_ for newly emerging diseases by adjusting the model with small portion of real data.*”

We apologize for unclear explanation on experimental settings and the synthetic data generation procedure. Please see **Response 2.3** for the more thorough discussion on experimental settings.

For better elaborating the generating procedure of the synthetic dataset, we have added a subsection about the **SEIR Transmission Model and the Ct Value Model** on the section of **Materials and methods**, which is presented as follows (**Line: 194**) :

**“*SEIR Transmission Model and the Ct Value Model***

*We adopt the agent-based stochastic Susceptible-Exposed-Infectious-Removed (SEIR) transmission model [57] and the Ct value model [25] to produce the synthetic dataset. The SEIR transmission model classifies the population into four types: susceptible (S), exposed (E), infectious (I) and removed (R). Therein, the susceptible state represents an individual who is not infected but can be infected, the exposed state represents an individual who is infected but not infectious, and the infectious state represents an individual who is infected and can infect susceptible individuals. Lastly, the removed state represents an individual who has recovered from the infectious state and can not be infected again. The flow of these states is presented in Fig 1A.*

*We employ the Ct value model reported in [25] to simulate the evolution of viral load (characterized by the Ct value) within host since the infection. In specific, we assume that the observed Ct value a days after infection, Ct(a), is represented by Eq. (S1) in S1 Supplementary Methods (1. Parameters of the Transmission Model). As shown in S1 Fig, the trajectories of Ct values capture the variation resulting from both swabbing variability and individual-level differences in viral kinetics, as shown in S1 Fig.*

*The parameters used to simulate both the SEIR model and the Ct value model are listed in S1 Table in S1 Supplementary Methods (1. Parameters of the Transmission Model).*”

We also have revised the steps to generate the synthetic data, which is listed as follows (**Line: 211**):

**“*Synthetic Datasets.*** *The synthetic dataset are generated with the same framework but with different transmission parameters. For each simulation, the data is produced with following three steps: 1) On each day of the simulation, we simulate the SEIR transmission model with one time step (i.e., one day) and produce the newly infected individuals at day t; 2) A subset of individuals are sampled on each day of the simulation determined by the detection scenarios described at the subsection of Impact of Detection Rates on Performance in Results. 3) For each individual i sampled on day a, a Ct value Ct(a-t) is generated according to the Ct value model. Therein, a-t represents the infection time of individual i, which is described in S1 Supplementary Methods (1. Parameters of the Transmission Model). To be noted that, at step 1), the micro-transmission chains including the time of each infected individual, and his/her infectees are kept, which were used to calculate the time-varying effective reproduction number R_t_. The details about the calculation are presented in S1 Supplementary Methods (2. Calculation of R_t_ based on Micro-transmission Chains).*”

2.1 The proposed Ct-Transformer model is designed to estimate time-varying effective reproduction numbers, while effective reproduction number can be defined in two ways—instantaneous reproduction number and case reproduction number Gostic et al. (2020). Does the proposed model focus on one of them or is it able to estimate both? It’s worth clarifying the scope of contribution at the beginning and, if necessary, illustrating both types of effective reproduction number estimates.

We apologize for the lack of clarity. The proposed model developed in this work focuses on estimating the case reproduction number. We have revised the first paragraph of **Introduction** to clarify the definition of time-varying effective reproduction number (**Line: 7**):

“*In specific, the time-varying effective reproduction number R_t_ can be defined in two ways: the instantaneous reproductive number and the case reproductive number [7]. Therein, the case reproduction number is defined as the average number of secondary cases generated by an infectious individual infected at time t [8], which is used in this work.*”

As the instantaneous reproduction number is derived from the current incidence of new infections and the distribution of generation intervals with the renewal equation, which exhibits some uncertainties when fitting with the data and also suffers from much computational cost. We regarded the case reproduction number as the time-varying effective number in this work, as it can be directly counted by keeping the micro-transmission chains when simulating the outbreaks. This is a limitation of the proposed Ct-Transformer. Thus, we have added the following sentence in the **Discussion part** (**Line: 495**):

“*Lastly, there are two kinds of definition on time-varying effective reproduction number: the instantaneous reproduction number and the case reproduction number. The former focuses on the short time windows and very sensitive to immediate changes in the transmission, while the latter reflects more on the actual transmission rather than the potential. Although the proposed Ct-Transformer performs well in estimating the case reproduction number, whether it will be effective in estimating the instantaneous reproduction number left for further investigations.*”

2.2 The authors used two (ER and SF) contact networks with different values of R_0_ in simulation. The networks help with representing the spread of epidemic among population following different contact patterns. What are the main differences between the two networks in terms of the contact patterns they can generate that make it worth while considering both rather than one of them? The authors also mentioned “degree” for the networks in line 87, but did not interpret it. An interpretation on what the “degree” level means to the epidemic transmission using the networks can be helpful to clarify the confusion. When R_0_ is first mentioned in line 87, it’s not defined. Does R_0_ represent Rt at time t = 0 or, by convention, the basic reproduction number?

We apologize for lack of clarity about why two kinds of networks are included in the analysis. Firstly, the ER network is a classical network model used as a theoretical baseline to study the spread of disease on networks. It characterizes the homogenous contact patter of population, which is a huge assumption to approximate the real contact network. Then the SF network is proposed to characterize the heterogenous contact pattern of the real contact network, where a small number of nodes in the SF network have a very high number of connections, while most other nodes have relatively few connections. Secondly, different propagation phenomenon can be observed when the same infectious disease respectively spreads on the ER network and SF network. For example, we found the R_t_ keeps the constant with time when the simulated outbreaks occur in ER network, while the R_t_ increases first and then decreases with time when the simulated outbreaks occurs in SF network, as shown in Fig R1. Besides, although both the ER and SF networks share the same average degree and the same transmission rate of the infectious disease. We observed that the R_t_ starts at a higher value in SF network than in ER network, this could be explained that there are the hub nodes in the SF network.Therefore, both the ER and SF networks are included to generate the synthetic data on the same infectious disease spreading in the population of different contact patterns, which is used to test the robustness of the proposed Ct-Transformer.

“***Fig R1****. The time-varying reproduction number R_t_ obtained in two single simualtions of tramission model on ER and SF networks, respectively. Therein, R_0_ is set as equal to 1.8.*

We also apologize for the lack of clarity on the concepts firstly presented in the manuscript. We have double-checked and corrected and added the detailed introduction of these in **the Data** (**Line: 223**) :

“*We consider multiple scenarios by modeling outbreaks on two distinct types of contact networks: the Erdős-Rényi (ER) network [58] and Scale-Free (SF) network [59]. The ER network is a random graph where edges between nodes are formed independently and randomly, and is used as a theoretical baseline to study the spread of disease on networks. The degree of a node, i.e., the number of neighbors or edges connected to a node in the ER network, approaches a Poission distribution, which is relatively narrow and most of nodes in the ER network having similar degrees. Thus, the ER network is used to characterize the homogenous contact pattern of population. In contrast, the SF network is developed to characterize the degree distribution of real networks, which usually follows the power law. It means that a small number of nodes in the SF network have a very high number of connections, while most other nodes have relatively few connections. We set the average degree of both type of contact network equal to 10 and simulate the outbreaks with different value of R_0_ (the average number of secondary cases generated by a typical infectious individual over the entire course of the infectious period in a fully susceptible population) to represent the modeled epidemic with different transmission abilities.*”

2.3 The authors provided a thorough discussion on the parameters in the proposed method, while the parameters used in the alternatives in the experiments are not completely listed. A complete discussion on the choice of parameters in the alternative methods in the experiments can make the experimental settings more complete and choosing the parameters to maximize the performance of alternative methods can make the experimental results more plausible.

We apologize for the lack of clarity regarding the process of searching the optimal hyperparameters in the alternative deep learning models. In the previous version, we applied a grid search approach to determine the optimal hyperparameters for these models. In this revised version, we have updated and clarified the method used to determine the optimal hyperparameters for all deep learning models (**Line: 285**):

“***Hyperparameter Selection****. A grid search approach [63] is employed to determine the optimal hyperparameters for the deep learning models. The specific tuning spaces and the best values for each hyperparameter of the Ct-Transformer are presented in Table 2. Meanwhile, the explored tuning spaces and the best hyperparameter values for all deep learning models are provided in S5 Table in the S1 Supplementary Methods (4. Hyperparameters of Deep Learning Methods). The hyperparameters yielding the minimum validation loss for the Ct-Transformer are used in subsequent experiments to estimate R_t_.*”

We have taken the suggestion of the reviewer and have added the table, i.e., *S5 Table on S1 Supplementary Methods (4. Hyperparameters of Deep Learning Methods),* which presents all the parameters used in the alternative deep learning models in this revised version.

2.4 In experimental results in Fig 3 in the manuscript and Figure S2_Fig in the Appendix, the R_t_ estimates start from (around) 20 days since start of outbreak. An explanation on why the first few days, 20 days specifically, are excluded from estimation or visualization can be useful for a better understanding and make the section more self-contained. Consequently, interpretation (and math notations, if necessary) on whether the accuracy of estimation is measured only after 20 days since start of outbreak can be helpful as well. Additionally, a brief explanation in the figure captions can make the figures more self-contained.

We apologize for the lack of clarity and for omitting the starting point in Fig 3 and S2 Fig. We begin using the proposed model and other deep learning models to estimate R_t_ once the number of cases in the simulated population exceeds fifteen, aiming to minimize MSE loss during model training. As shown in the Fig R2, we observed that the MSE loss is significantly higher at the very early stage of epidemic, indicating that the estimated results at that time are unreliable.

“***Fig R2****. Performance of the supervised Ct-Transformer on ER network*. *Panels (A) and (B) display the R_t_ estimations by the Ct-Transformer where R_0_ is set to 1.8 and the corresponding MSE loss, respectively.*”

Therefore, we begin estimating R_t_ once there are 15 cases in the simulated population. Due to the stochastic nature of the agent-based SEIR transmission model, the number of days required to reach 15 cases varies across simulations. However, as the reviewer noted, it typically takes around 20 days in these figures. We have revised the captions for Fig 3 and have added the necessary explanations (**Line: 311**):

“***Fig 3****. The performance of the supervised Ct-Transformer on two stochastic simulations on the ER network. Panels (A) and (B) display the R_t_ estimations by the Ct-Transformer and other alternative models for simulations where R_0_ is set to 1.8 and 3.4, respectively. To be noted that, all models begin estimating R_t_ when there are fifteen cases in the simulated population. The ground truth values are derived from the micro-transmission chain of the two stochastic simulations, based on the definition of the case reproduction number. The estimation results of EpiEstim (dotted yellow line), ViroSolver (dotted green line), and TFT (orange line) are presented as the representative alternative methods for incidence-based, Ct-based statistical, and Ct-based deep learning approached, respectively*.”

Minor comments

A more thorough literature review can make the paper more self-contained. Readers can also benefit from a more detailed explanation on parameters and clarification of a few abbreviations. Details are listed as follows.

2.5 In line 66, the authors stated their method outperforms other alternatives. A brief discussion on the specific aspects, e.g., accuracy of Rt estimation, that the proposed method improves upon compared to others can make the point more clearly stated

Thank you for this suggestion. As suggested, we now have added this information at the end of the **Introduction** (**Line: 75**):

“*The main aim of this study is to estimate the case reproduction number from the time-varying distribution of Ct values based on deep learning models. In order to better capture the long-range dependencies and extract the temporal features, we propose the Cycle Threshold-based Transformer (i.e., Ct-Transformer) with the canonical components, including the patching layer, the gated recurrent unit network and the multi-head attention layer. The detailed descriptions of the above components in the architecture of Ct-Transformer are presented in Materials and Methods. To be noted that, both the supervised learning and the self-supervised learning of Ct-Transformer are developed. Therein, the supervised learning of Ct-Transformer is trained on a large number of datasets with labels, while the self-supervised one can be trained on the datasets without labels and fine-turning the pre-trained model on small portion of labeled data, which finally both of them can be used to estimate R_t_. By evaluating the Ct-Transformer on the synthetic datasets, we find the supervised learning of Ct-Transformer performs best in all Ct-based deep learning and statistical methods. More in detail, the supervised learning of Ct-Transformer achieves a 31.1% reduction in MAE and a 27.0% reduction in RMSE, compared with other cycle thresholds based deep learning methods. We also find the proposed Ct-Transformer is robust to both the limited and the time-varying detection resources. It achieves similar performance or better performance in the situations with 25% detection rate compared with the traditional incidence-based R_t_ estimation method in the situations with 100% detection rate. Lastly, we pre-train the self-supervised learning of Ct-Transformer on synthetic datasets, and then fine-turn the pre-trained model on the Hong-Kong COVID-19 data. We obtain a 24% reduction in MAE compared with the result reported in [31].*”

2.6 The literature review in lines 11–21 can be enhanced by citing relevant literatures on accurate time-varying effective reproduction estimation using generation time distribution, e.g., Abbott et al. (2020); Gressani et al. (2022); Liu et al. (2023); Parag (2021), which are alternatives to EpiEstim.

Thank you suggesting these relevant papers. As suggested, we have broaden our literature review to include the suggested studies. (**Line: 12**):

“*Most existing studies [1,10-17] have adopted the statistical methods to estimate R_t_ based on the time series of daily symptomatic cases, hospitalizations and death numbers. Wallinga and Teunis [10] propose a likelihood-based method (WT method) to estimate R_t_ based on the daily number of reported cases and the generation interval distribution. The estimation results of this method may show substantial variation in a short period when the data aggregation time step is small. As an improvement, Cori et al. [13] develop the EpiEstim, a generalized R toolkit that estimates R_t_ based on Bayesian inference. The EpiEstim provides precise posterior distribution of R_t_ and has been employed to monitor several recent outbreaks [17-20]. Gressani et al. [14] propose the EpiLPS, which can smooth the epidemic curve and allows to obtain accurate estimates of R_t_. Liu et al. [15] propose a discrete spline-based approach rtestim and produce a locally adaptive estimation results for R_t_. Parag et al. [16] construct a recursive inference algorithm and develop the EpiFilter. This method unifies the WT method and EpiEstim and largely resolves their edge-effect issues. These methods typically require the assumption of the generation interval distribution (i.e., the time interval between the infection time of the infector and her/his infectees), which is hard to acquire at the early stage of the emergent epidemic [7,21]. Further, the aforementioned time series data is sensitive to detection resources, latent periods, and reporting delays [22-24].* ”

2.7 The abbreviation “GNN” is not defined when it’s first mentioned in line 52

We apologize for the lack of clarity regarding the abbreviation “GNN” when it was first introduced. The sentence has been revised as follows (**Line: 60**):

“*Davahli et al. [40] develop two types of Graph Neural Networks (GNN) models [44], which are designed to handle graph-structured data, to estimate R_t_ using COVID-19 cases from 22 January 2020 to 26 November 2020 in the United States.*”

2.8 The abbreviation “LSTM” is not defined when it’s first mentioned in line 54

We apologize for the lack of clarity regarding the abbreviation “LSTM” when it was first introduced. The sentence has been revised as follows (**Line: 55**):

“*The early notable deep learning models include Recurrent Neural Networks (RNNs) [39] and Long Short-Term Memory (LSTM) networks [39]. Therein, the architecture of RNNs is designed to extract temporal feature from sequence data, while the LSTM, as an advanced form of RNNs, is built for capturing long-term dependencies in sequence data.*”

2.9 In line 105, Ct value at each time is defined as a vector of dimension M. What does the dimension refer to? Is it a hyperparameter of users’ choice?

We apologize for unclear definition of dimension M. The dimension M refers to the number of Ct variables, including the mean, skewness and distribution of Ct values. In the discussion in the appendix, the dimension M refers to a hyper-parameter, which is used to explore the most suitable combination for model learning. We have made additions in the subsection of The Supervised Learning of Ct-Transformer (**Line: 109**):

“*The dimension M refers to the number of Ct variables, including the mean, skewness and distribution of Ct values. Detailed exploration of dimension M is shown in S1 Supplementary Methods (3. Ct Variables in the Synthetic Datasets).*”

2.10 The abbreviation “RNN” is not defined when it’s first mentioned in line 135

We apologize for the lack of clarity regarding the abbreviation “RNN” when it was first introduced. The sentence has been revised as follows (**Line: 55**):

“*The early notable deep learning models include Recurrent Neural Networks (RNNs) [39] and Long Short-Term Memory (LSTM) networks [39]. Therein, the architecture of RNNs is designed to extract temporal feature from sequence data, while the LSTM, as an advanced form of RNNs, is built for capturing long-term dependencies in sequence data.*”
